# Characterization of faecal microbial communities of dairy cows fed diets containing ensiled *Moringa oleifera* fodder

**DOI:** 10.1038/srep41403

**Published:** 2017-01-30

**Authors:** Jiajie Sun, Bin Zeng, Zujing Chen, Shijuan Yan, Wenjie Huang, Baoli Sun, Qian He, Xiaoyang Chen, Ting Chen, Qingyan Jiang, Qianyun Xi, Yongliang Zhang

**Affiliations:** 1 College of Animal Science, Guangdong Provincial Key Laboratory of Agro-Animal Genomics and Molecular Breeding, National Engineering Research Center for Breeding Swine Industry, South China Agricultural University, Guangzhou, Guangdong 510642, China; 2 College of Forestry and Landscape Architecture, Guangdong Engineering & Research Center for Woody Fodder Plants, South China Agricultural University, Guangzhou, Guangdong 510642, China; 3 Agro-biological Gene Research Center, Guangdong Academy of Agricultural Sciences, Guangzhou 510640, Guangdong Province, China

## Abstract

*Moringa oleifera (M. oleifera*) is a remarkable species with high nutritional value and good biomass production, which can be used as livestock fodder. In this study, we examined changes in the faecal microbiota of thirty dairy cows in response to alternative *M. oleifera* diets and their effects on nutrient digestion, milk traits and the faecal concentrations of short-chain fatty acids. No differences in milk yield and constituents were found between the control and the *M. oleifera* alternative groups. Cows fed *M. oleifera* silage had lower dry matter digestibility, as well as the propionate and isovalerate concentrations in *M. oleifera* treated group. Using 16S rDNA gene sequencing, 1,299,556 paired-end reads were obtained. Clustering analysis revealed 13 phyla and 93 genera across all samples. *Firmicutes* and *Bacteroidetes* were the co-dominant phyla. Ten taxa displayed a significant difference in response to the high *M. oleifera* diet. In addition, strong correlations between *Akkermansia* and *Prevotella* with milk yield and protein indicated that some bacterial groups could be used to improve milk traits. Our results provided an insight into the microbiome-associated responses to *M. oleifera* in livestock diets, and could aid the development of novel applications of *M. oleifera*.

Currently, many countries have banned the use of animal by-products and waste materials as a source of proteins in diets for livestock to ensure the consumer safety of animal products^1^. In addition to processed animal proteins, the availability and price of plant protein sources for animal feed, such as the soybean meal used commonly for protein supplement in animal diets, is a serious issue for animal producers^2^, especially for small farm stakeholders. Consequently, there is a need for alternative, cheaper ingredients with high protein contents and balanced amino acids profiles. Historically, some trees and browse species have been chosen as livestock fodder, especially during dry periods; for example, poplar and willow^3^, *mulberry*^4^, *Leucaena leucocephala, Bauhinia purpurea*^5^, *Caragana*^6^, and *Moringa oleifera*^7^. Among these potential candidates, *M. oleifera* is the most useful tree as an animal feed supplement, because its leaves are highly nutritious, with excellent palatability, digestibility, and contain a balanced chemical composition of proteins and minerals^7^. The protein content of *M. oleifera* generally ranges from 15% to over 30% dry matter (DM), which depends on the stage of maturity and on the fodder’s respective proportions of leaflets, petioles, and stems; the latter being much poorer in protein. Recently, researchers have explored *M. oleifera* cultivation practices and its utilization as livestock fodder to improve animal production in goats^8^, sheep^9^, and cows^10^. Previous studies using fresh *M. oleifera* foliage^11^ or silage^12^ for dairy cattle have shown improvements in, or at least no affects on, live weights, milk yield, and composition compared with traditional diets. However, to date, there has been little research investigating the effect of alternative *M. oleifera* diets on the dynamics of intestinal microbial communities in dairy cows. In addition, the microbes’ metabolic processes have a direct influence on the host’s gut health and nutrient availability^13^, which should be considered when formulating diets with unconventional ingredients.

The mammalian gastrointestinal tract has been estimated to contain 500–1000 microbial species and to comprise approximately 10^14^ microbes that constantly interact with the host and other members of the microbial community^14^. Culture-dependent methods are inadequate to determine gut microbial profiles because only 1% of intestinal microbes with high copy numbers are culturable^15^. Recently, in-depth descriptions of the gut microbiota have been produced mainly using high throughput DNA sequencing of 16S rRNA genes^16^. Hence, in the present paper, we aimed to characterize the faecal microflora of dairy cows by sequencing their 16S rRNA genes, and to assess quantitatively whether the underlying faecal microbial composition and structure changed in response to ensiled *M. oleifera* diets.

## Results

### 16S rRNA gene sequencing and annotation analysis

**Figure 1:**
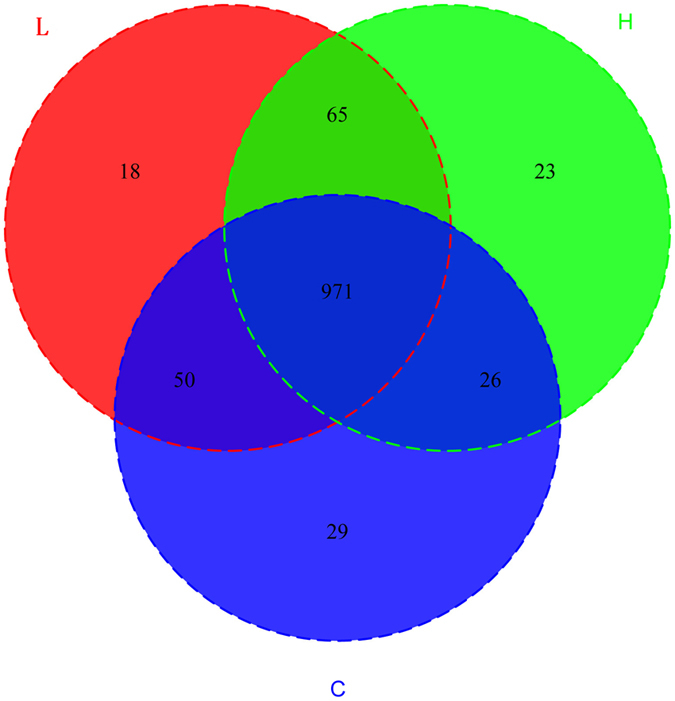
Common and specific OTU distribution of the faecal microbiota among three groups. Note: Blue represents the no *M. oleifera* group (N = 8); Red represents the low *M. oleifera* group (N = 12); Green represents the high *M. oleifera* group (N = 10).

**Figure 2:**
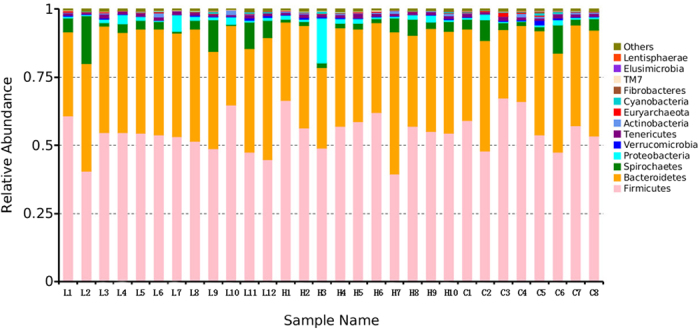
Phylum-level microbial compositions in the faecal microbiome. Note: The x-axis is labelled with the sample names, and y-axis represents the relative abundance of each phylum. C represents the no *M. oleifera* group; L represents the low *M. oleifera* group; H represents the high *M. oleifera* group.

Fresh faecal samples were collected from eight dairy cows fed with no *M. oleifera* (C), twelve cows fed a low *M. oleifera* diet (L), and ten cows fed a high *M. oleifera* diet (H). After DNA extraction, the hypervariable V3–V4 regions of the 16S rDNA were enriched in each sample, and the subsequent high-throughput analysis generated a 1,299,556 paired-end Illumina reads representing more than 435.95 megabases (Mb). On average, each sample produced approximately 43,318 joined tags, which were assembled by PandaSeq v2.8 (min = 34,706, max = 58,766). Over 85.52% ± 5.33% of the total joined tags from each sample passed quality control and were processed for further analysis (Table S2). Clustering of operational taxonomic units (OTUs) analysis of the high-quality tags yielded 1,182 unique OTU candidates at 97% sequence similarity, and 971 candidates that were shared across all samples were defined as core OTUs. The core OTUs comprised approximately 82.15% of the total candidates, while only 29, 18, and 23 OTUs were identified uniquely in the C, L and H groups, respectively (Fig. 1). We annotated all these OTU tags to the Greengenes database, and found that 99.43% ± 0.20% sequences could be aligned at the phyla taxonomic level, while 99.01% ± 0.57%, 98.99% ± 0.58%, 80.84% ± 2.73%, 35.35% ± 5.48%, and 1.27% ± 0.73% of the annotated OTUs were assigned at the class, order, family, genus, and species level, respectively (Table S3). In addition, the microbial diversity in the faeces of dairy cows was assessed using the quantitative insights into microbial ecology (QIIME) pipeline, based on the OTU annotation, which identified 13 phyla (Fig. 2). The most abundant phylum in the faeces of dairy cows was *Firmicutes*, which accounted for approximately 54.59% ± 7.07% of all sequences, followed by *Bacteroidetes* (36.25% ± 5.37%), *Spirochaetes* (4.16% ± 3.82%), and *Proteobacteria* (1.87% ± 3.04%) (Table S4A). Among the 93 genera detected, ten genera had a relative abundance greater than 1.0%, such as *5-7N15, Treponema, CF231, Ruminococcus, Prevotellaceae-Prevotella, YRC22, Oscillospira, Coprococcus, Clostridium*, and *Roseburia* (Table S4B).

### Microbial diversity in the faeces of dairy cows

**Figure 3:**
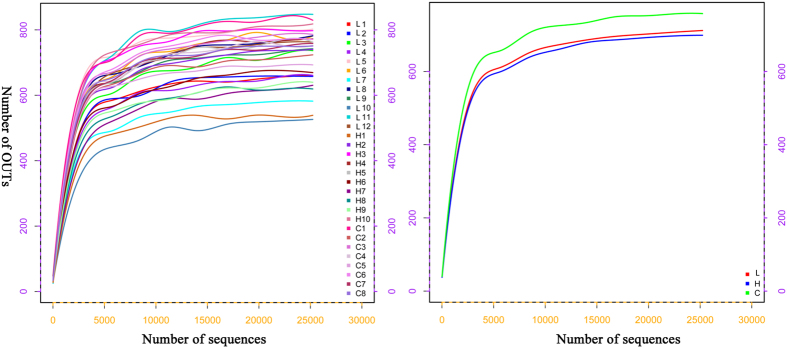
Rarefaction curve analysis in the faecal microbiome. Note: (**A**) Rarefaction curves between individuals (left) and groups (right). C represents the no *M. oleifera* group (N = 8); L represents the low *M. oleifera* group (N = 12); H represents the high *M. oleifera* group (N = 10).

To compare the alpha-diversity (within-sample diversity or an estimate of species richness and evenness) of each sample with differing sequence counts/sampling efforts directly, we rarefied the data (i.e., randomly picked an equal number of sequence across the samples) using QIIME. Rarefaction curve analysis indicated that the sequencing depth was sufficient to wholly capture the diversity present, as displayed by the microbial diversity index Chao1 (Fig. 3). In addition, we further used Chao1, Shannon, observed species, and phylogenetic diversity_whole tree estimator between different groups to evaluate the faecal diversity of dairy cows affected by *M. oleifera* treatments (Table S5). Although there was a trend towards decreased alpha diversity in the *M. oleifera* groups compared with the control, these differences did not appear to significantly affect the species-level microbial diversity using the three measures (Chao 1 index, observed species, and phylogenetic diversity_whole tree). For example, the mean community richness, as estimated by the Chao 1 index, was similar between the C, L, and H groups, comprising 760.84 ± 14.39, 714.79 ± 27.41 and 703.30 ± 29.55, respectively. In addition, the Shannon diversity index, which is used commonly to characterize species richness and evenness in a community, tended to be lower in the L and H groups compared with group C (7.54 ± 0.07 versus 7.32 ± 0.10 and 7.21 ± 0.11, respectively; *P* = 0.098).

### *M. oleifera* alternative diets altered the composition of faecal microbiota

**Figure 4:**
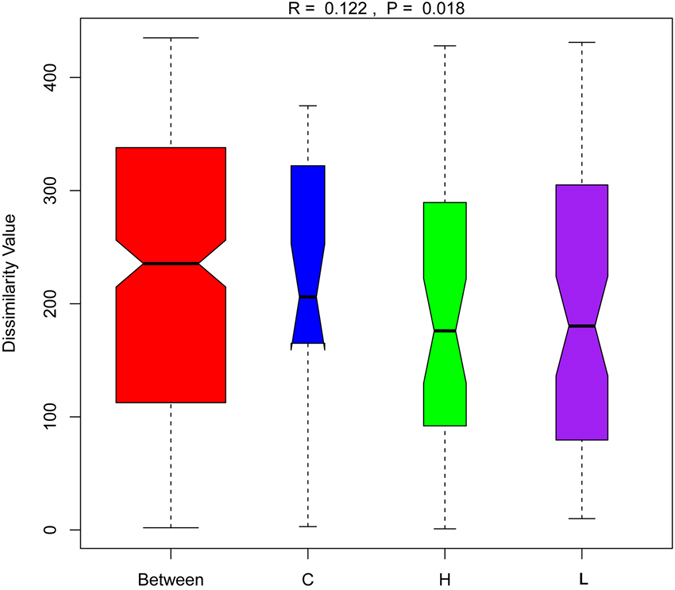
Anosim analyses of the significant differences between groups at genus-taxa level. Note: Anosim, analysis of similarity. The R-value ranged from −1 to 1, and an R-value > 0 that showed the significant differences in the between-group population compared those in the within-group population. The *P*-values < 0.05 indicate a significantly different level between groups.

To test for dissimilarities between samples collected from different *M. oleifera* diet groups, a global one-way analysis of similarities (ANOSIM) of Bray–Curtis similarities was performed. This analysis suggested that there were large and significant differences between groups (global R = 0.122, *P* = 0.018) (Fig. 4). Pairwise ANOSIM analyses between different groups are shown in Table 1. Among the pairwise correlations, significant differences were identified between the C and H groups (*P* = 0.024). In addition, to further confirm whether alternative *M. oleifera* diets were of greater specific benefit for the faecal microbiome of dairy cows, we also performed a multi-response permutation procedures (MRPP) analysis in the within and between-group populations (Table 1). The differences in the between-group homogeneity were higher than those for the within-group population, with an A-value > 0, and the change degree between group C and H reached statistical significance (*P* = 0.018), as did that between the C and L groups (*P* = 0.03). Similar results were obtained using non-metric multi-dimensional scaling (NMDS) analysis, which revealed that the microbial community compositions were significantly different between group C and group H (Figure S1). The complementary analyses using ANOSIM, MRPP and NMDS provided similar results; therefore, the differences in the microbial composition (relative abundance) of faecal microbiome between the C and H groups were compared using the Metastat method with Fisher’s exact test^17^. Ten genus-level taxa displayed a significant difference in response to the high *M. oleifera* diet. The high *M. oleifera* diet increased the proportion of *Methyloversatilis, Caulobacter, Ruminococcus, Peptococcaceae_ rc4-4, Ralstonia,* and *Devosia* remarkably, and decreased the abundance of *Coprococcus, Epulopiscium, Bacteroides*, and *Fibrobacter* significantly, compared with group C (Table S6A). At the OTU (species) level, 180 OTUs showed a significant difference in their abundance between the C and H groups, with 60 and 120 candidates showing increases and decreases in the H group, respectively (Table S6B), which belonged to 28 representative genus taxa. Among them, 15 OTUs were from the genus *Ruminococcus*, especially the species *bromii* and *gnavus*, which responded significantly to the alternative *M. oleifera* diet.

### Apparent nutrient digestibility and milk traits of dairy cows and their correlation with the faecal microbiota

**Figure 5:**
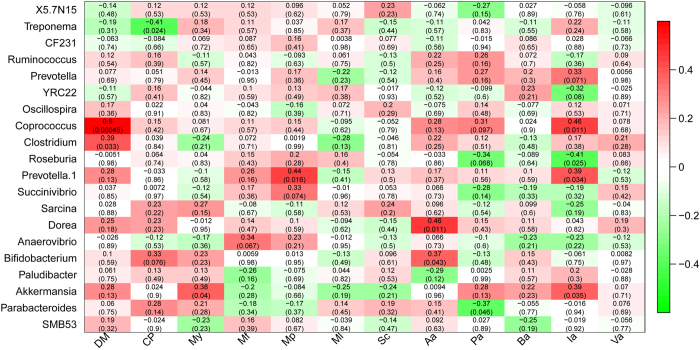
Correlation analyses of genus taxa with the apparent digestibility, milk traits and faecal SCFA concentrations. Note: Each cell contains the corresponding correlation and *P*-value. The table is colour-coded by correlation according to the colour legend. DM: dry matter digestibility; CP: crude protein digestibility; My: milk yield; Mf: milk fat; Mp: milk protein; Mi: lactose, Sc: somatic cell count; Aa: acetic acid; Pa: propionic acid; Ba: butyric acid; Ia: isovaleric acid; Va: valeric acid.

No convincing differences in milk yield and its constituents (protein, fat, lactose, and somatic cell count) in dairy cows were found between the control and the *M. oleifera-*fed groups (*P* > 0.05). Feeding *M. oleifera* silage to dairy cows dramatically decreased the apparent digestibility of the DM (*P* < 0.01), but no apparent effect were observed on the digestibility of crude protein (CP) (*P* > 0.05) (Table 2). To further identify the phyla that correlated significantly with the nutrient digestibility and milk traits of dairy cows, we used the Pearson correlation test. We observed that the decreased *Fibrobacteres* abundance was directly and significantly associated with a modest decrease in the apparent digestibility of DM (Pearson’s r = 0.470, *P* = 0.009). Milk somatic cell count displayed a strong positive (Pearson’s r = 0.547, P = 0.005) and negative correlation (Pearson’s r = −0.497, P = 0.011) with the relative abundances of the bacterial families *Firmicutes* and *Bacteroidetes*, respectively (Table S7). At the genus level, the bacterial abundances of *Coprococcus* and *Clostridium* correlated positively with the apparent digestibility of DM, and the bacterial abundances of *Treponema* were significantly negatively correlated with the apparent digestibility of CP (Fig. 5). In addition, there were strong correlations between two bacterial families, *Akkermansia* and *Prevotella*, with milk yield and protein contents across all samples.

### Analyses of short-chain fatty acids (SCFAs) in the faeces of dairy cows and their correlation with the faecal microbiota

The major SCFAs observed in the faeces of dairy cows were acetate, propionate, butyrate, and valerate as well as isovalerate (Table 3). Comparison of SCFA concentrations between different tested groups revealed that all selected SCFA candidates appeared a general downtrend in response to the *M. oleifera* diet, and the propionic (*P* = 0.029) and isovaleric (*P* < 0.01) acids were substantially and significantly lower in faeces of dairy cows fed *M. oleifera* silage than in those given the basic diet. In details, feeding *M. oleifera* silage to dairy cows dramatically decreased the propionate concentration in H population (P = 0.013), as well as in L group (P = 0.027). Compared with the C group, the faecal concentrations of isovalerate were lower in L and H groups; and with the increase of the alternative proportion of ensiled *M. oleifera* fodder, the isovalerate concentration also sharply reduced in H group compared with L group (P = 0.004). In addition, the correlation analyses of genus taxa with the faecal concentrations of SCFAs were tested (Fig. 5), and the results showed that the gut microbial communities of dairy cows were highly related to isovaleric acid. Briefly, isovaleric acid displayed a strong positive correlation with the relative abundances of the bacterial taxa *Prevotella (P* = 0.071), *Coprococcus (P* = 0.011), *Prevotella.1 (P* = 0.034) and *Akkermansia (P* = 0.035), and negatively associated with *Roseburia* abundance (P = 0.025). For other SCFAs, *Dorea (P* = 0.011) correlated positively with acetate concentration, while *Parabacteroides (P* = 0.046) correlated negatively with propionate, respectively.

## Discussion

In this study, 1,103,963 high quality valid tags were obtained across all samples, and the sequencing size of each sample ranged from 30,246 to 43,732, which were higher than those in previous studies of mouse^18^, human^19^, pigs^20^, and dairy cows^21^. Rarefaction curves analysis of the Chao1 index indicated that this sequencing depth was sufficient for further analysis. Clustering analysis revealed that *Firmicutes* and *Bacteroidetes* were the most dominant phyla, regardless of diet, representing approximately 90.84% ± 4.22% of the total sequences. These results were similar to previous observations in porcine and human faecal samples. Niu *et al*.^22^ reported that the bacterial communities of porcine faeces primarily comprised *Firmicutes* and *Bacteroidetes*, which accounted for approximately 80% of the total sequences. Lamendella *et al*.^23^ further confirmed the observation that most of the beneficial bacteria in porcine gut microbiome were *Firmicutes* and *Bacteroidetes*. In addition, these two phyla comprised over 90% of the known phylogenetic categories, representing the dominant gut microbiota in the human intestinal tract^24^. In addition to the expression level, the functions of these two phyla taxa require further study.

Recently, diet and nutritional status have emerged as major factors affecting the structure and function of the gut microbiome. In porcine faecal specimens, gut microbiomes were associated significantly with the level of dietary fibre solubility^25^. In mice, a whole-wheat supplemented diet increased the *Bacteroidetes*/*Firmicutes* ratio of faecal microbiota and decreased the abundances of the families *Enterococcaceae, Turicibacteraceae,* and *Ruminococcaceae*^26^. Similarity analysis revealed that the relative proportion of *Bacteroidetes* decreased in obese people, which correlated strongly with a low-calorie diet^27^. In the present study, feeding *M. oleifera* silage to dairy cows also resulted in significant shifts in the composition and structure of the faecal microbial community compared with the control diet. At genus-level taxa, the abundance of *Ruminococcus spp.* that responded to alternative *M. oleifera* diets increased obviously in the faeces of dairy cows, which was similar to previous reports in horse^28^ and cattle^29^ faeces. A previous study reported that *Ruminococcus* mainly inhabits the gastrointestinal tract of ruminants^28^, and are known for their ability to degrade a wide range of plant polysaccharides, including cellulose^30^. Therefore, a hypothesis was proposed that alternative *M. oleifera* diets likely increased the available fibre; however, *Ruminococcus* could compensate for this effect. More detailed determinations are needed to resolve this issue. By contrast, the mean abundance of *Coprococcus* in the faecal microbiome of dairy cows was 1.36% ± 0.33%, and decreased by 28.52% in the high *M. oleifera* group (*P* < 0.05). The genus *Coprococcus spp*., which is affiliated with the family *Lachnospiraceae*, was correlated positively with the production of SCFAs in the human intestine^31^. The health-promoting functions of SCFAs include their roles as nutrients for the colonic epithelium and modulators of colonic pH, and a decrease in SCFAs resulted in damage to the normal function of the colonic epithelium^32^. In our paper, alternative *M. oleifera* diets obviously decreased the faecal concentrations of SCFAs, especially for Propionic and Isovaleric acids. The correlation analyses of genus taxa with the faecal SCFAs also revealed that *Coprococcus* correlated positively with isovalerate concentration (P = 0.011). Hence, the *M. oleifera*-induced decreases in *Coprococcus* might be involved in intestinal pathogenesis by affecting the function of isovalerate, which supported the results of Dicksved *et al*.^33^. That study showed that *Coprococcus* were present in higher abundances in early-weaned piglets compared with suckling piglets, also suggesting the functions of *Coprococcus* in supplying energy to enterocytes and inhibiting intestinal inflammation^33^.

Various reports have highlighted the importance of *M. oleifera* as a cost-effective protein source supplement for livestock fodder that can be used in ruminant feeding. Mendieta-Araica *et al*.^10^,^12^ reported that feeding fresh *M. oleifera* foliage to dairy cows caused higher digestibility of DM and CP, but no significant differences were found in milk yield and composition. Goats fed *M. oleifera* leaves markedly increased their DM intake and the digestibility of most nutrients increased compared with the control diets. Milk yield, energy, and nutrient outputs were also increased by replacing sesame meal with *M. oleifera* leaf meal in lactating goats^11^. In addition, similar performances were observed in goats fed *M. oleifera* silage or hay, followed by fresh *M. oleifera*^34^. In contrast to these previous studies, in this study, feeding *M. oleifera* silage to dairy cows decreased the apparent digestibility of DM, but no additional effects were observed on the digestibility of CP. The discrepancies between the present and previous studies might have resulted from the different parts of *M. oleifera* plants used in the experiments (whole plant *vs.* leaves)^35^, different treatments (silage *vs.* fresh) or species (dairy cows *vs.* goat). Diets are mainly degraded by the gut microbiota^36^, and a number of microbials, such as *Bacteroides* and *Butyrivibrio*, are related to the apparent digestibility of nutrients in sheep^37^ and cows^38^, respectively. Coincidentally, the abundance of *Fibrobacteres* was positively correlated with the apparent digestibility of DM in the present study, as were *Coprococcus* and *Clostridium* at genus taxa level. In addition, the abundances of the phylum *Tenericutes* and the genus *Treponema* correlated negatively with the apparent digestibility of CP. However, to date, few microbiota have been reported to modulate nutrient digestibility in dairy cattle. Generally, milk production is a direct result of improved feed utilization, and our results showed a reduced tendency for milk yield, which agreed with the decreased digestibility of DM. Additionally, correlation analysis revealed that *Akkermansia* and *Prevotella* were associated strongly with milk yield and protein contents, indicating that some bacterial groups can be used to improve milk traits.

In conclusion, *M. oleifera* fodder can be used as a protein source in diets for lactating cows. The inclusion of *M. oleifera* meal decreased the apparent digestibility of DM, but no significant differences were found in milk yield and composition. Furthermore, the study investigated *M. oleifera*-induced shifts of metabolically active bacteria in the faeces of dairy cows, and showed that the variations of bacterial diversity of each individual were affected by less abundant bacterial components in the faeces. A correlation analysis of feed utilization and milk traits with gut microbiota revealed potential candidate taxa that might prove useful for future inoculation studies.

## Materials and Methods

### Animals, feeding and management

The animal feeding experiments were conducted at the Ranch of Xianquan Lake, in Guangzhou, Guangdong, China (Latitude: 23.13N, Longitude: 113.65E), from the 13^th^ of December 2015 to the 23^rd^ of January 2016 (Daily maximum temperature: 18.65 ± 2.72 °C). Sixty healthy Holstein cows were selected with similar ages, weights, parities, and lactation periods, and were randomly divided into three equal groups, termed no *M. oleifera* (fed a basal diet), low (25% alfalfa hay and 50% maize silage were replaced by *M. oleifera* silage, DM basis), and high (50% alfalfa hay and 100% maize silage were replaced by *M. oleifera* silage, DM basis) *M. oleifera* diet groups. The ingredients and nutritional composition of each diet group are shown in Table S1. All animals were fed *ad libitum* and had free access to water during the day except at milking time. All animal experimentation complied with the regulation of laboratory animal management and welfare approved by the Standing Committee of Guangdong People’s Congress, China. All experimental procedures were carried out with the approval of the ethics committee of South China Agricultural University.

### Sampling and chemical analysis

The Holstein cows were pre-fed with experimental diets for 10 days and were subsequently tested at the same time for 4 weeks. During the experimental periods, the dairy herds were milked robotically twice daily at an approximately 12 h interval. The yield from each individual was weighed and recorded. At the last day of the four-week experiment, 100 ml milk samples were collected in sterile vials and immediately refrigerated at 4 °C for fat, protein, lactose content, and somatic number analysis. During the last 3 days of the experiment, in each group, feed and faecal samples were analysed to estimate the apparent digestibility of DM and CP using the acid-insoluble ash (AIA) method^39^. In brief, the diets and faeces of each animal were collected daily and pooled equally into one sample per cow, and then the mixtures were dried, ground and passed through a 1-mm sieve before the chemical analysis. The digestibility values of the chemical components were calculated by the following equation: X digestibility = [1 − (diet AIA content/faecal AIA content) × (faecal X content/diet X content)] × 100%, X = DM or CP.

### SCFAs determination

The SCFAs extraction procedures for faecal samples were performed according to the previous study with some modifications^40^. Briefly, 50 μL internal standard solution (caproic acid-d3) and 1 mL of 0.005 M aqueous NaOH were added to 100 mg faecal samples. Then the samples were vortexed for 15 min, and centrifuged at 13,000 × g for 10 min. Next, a total of 500 μL supernatant solution was transferred into a 10 mL glass centrifuge tube followed by adding 150 μL water. For derivatization, 250 μL propanol/pyridine mixture solvents and 50 μL Propyl-chloroformate (PCF) were subsequently added to the glass tube, and intermittently vortexed for 10 min. After the derivatization, 300 μL hexane was applied for SCFA derivative extraction, and the extraction procedure was then repeated by adding 200 μL of hexane to the reaction mixture again. An aliquot of 150 μL and 100 μL SCFAs derivatives (the upper hexane layer) from the two steps was combined. Approximately 10 mg anhydrous sodium sulfate was used to remove the traces of water. By centrifugation at 13,000 × g for 5 min, 250 μL SCFAs derivatives were transferred to a glass vial prepared for GC-MS analysis.

A standard mixture containing seven authentic standards (acetic acid, propionic acid, butyric acid, isovaleric acid, valeric acid, caproic acid and caproic acid-d3) dissolved in 0.005 M aqueous NaOH were also prepared with the same procedure. Analysis was performed using an Agilent 7890A gas chromatography system coupled to an Agilent 5975C inert XL EI/CI mass spectrometric detector (MSD, Agilent Technologies, Santa Clara, CA). Helium was used as a carrier gas at a constant flow rating at 1 mL/min. The injection volume of the samples was 1 μL in split mode with a ratio of 50:1, and the solvent delay time was set to 2 min. The initial oven temperature was held at 50 °C for 2 min, ramped to 70 °C at a rate of 10 °C min^−1^, to 85 °C at a rate of 3 °C min^−1^, to 110 °C at a rate of 5 °C min^−1^, to 290 °C at a rate of 30 °C min^−1^, and finally held at 290 °C for 8 min. The MS was operated in the electron impact ionization mode at −70 eV, and the temperature of the front inlet and transfer line were set at 260 and 290 °C, respectively. Mass spectral data were collected in full scan mode from m/z 30–600. Raw GC–MS data files were converted to NetCDF files using the Agilent’s MSD Chemstation Data Analysis Application. Subsequently, the “Statistical Compare” function in ChromaTOF software (Leco Corp., St. Joseph, MO) was utilized to generate a data table with all the compound information from different chromatograms.

### DNA extraction, 16S rRNA gene amplicon preparation and sequencing

Approximately 100 g of mixed faeces were prepared and freeze-dried before DNA extraction using the method of Rius *et al*.^41^ DNA integrity was verified using a BioAnalyzer 2000 (Agilent, Palo Alto, CA, USA), and concentration was then determined using a Qubit fluorometer (Invitrogen, Carlsbad, CA, USA). The hypervariable V3–V4 regions (*Escherichia coli* position 341 to 806) of the 16S rRNA gene, which have been targeted frequently for interrogating bacterial communities, were directly amplified from 10 ng of total DNA using PAGE-purified Illumina platform-compatible adaptor oligonucleotides that contained sequencing primers, sample-specific barcodes, and 16S PCR primers (forward primer, 341F: CCTAYGGGRBGCASCAG; reverse primer, 806R: GGACTACNNGGGTATCTAAT). The PCR reaction included 1 U of FastPfu DNA Polymerase (TransStart, Haidian, Beijing, China) in a 20 μl reaction buffer, containing 0.2 μM primers, 0.25 mM dNTPs, 20 mM Tris-SO_4_ (pH 9.2), 40 mM KCl, 10 mM (NH_4_)SO_4_, 2 Mm MgSO_4_, and 2.5% Glycerol. PCRs were performed in an Eppendorf Mastercycler (Eppendorf, Hamburg, Germany) using the following conditions: 95 °C for 5 min; then 27 cycles of 95 °C for 30 s, 55 °C for 30 s, 72 °C for 45 s; and elongation at 72 °C for 10 min; then 10 °C until halted by user. Amplicons were purified using an Agencourt AMPure XP bead kit (Beckman Coulter Genomics, Danvers, MA, USA) and pooled using equal amounts of each PCR product. Finally, the library pool was sequenced using an Illumina MiSeq Reagent Kit (Illumina, San Diego, CA, USA) on an Illumina MiSeq sequencer.

### Bioinformatics and data analysis

Raw sequences were preprocessed using MiSeq Control Software (MCS) v2.4.1, and split based on sample-specific barcodes. The sequencing reads were removed if their lengths were shorter than 200 nt, their average quality score was < 25, and they contained more than one ambiguous base “N”, primer mismatches or errors in the barcodes. Finally, the processed pair-end (PE) reads were joined together using PandaSeq v2.8 to generate representative complete nucleotide sequences termed “Raw PE”, with default parameters^42^. *De novo* and reference-based chimera checking was then performed by blasting the sequences against the Gold database (http://drive5.com/uchime/gold.fa) based on the UCHIME Algorithm^43^ and raw PE tags that were characterized as chimeras were removed. After filtering of noisy sequences, the effective tags were uploaded into QIIME and analysed as described previously^44^. Briefly, sequences were clustered into OTUs using the UPARSE software^45^ with a minimum pair-wise identity of 97%. Each cluster was represented by its most abundant sequence, and representative OTU sequences were then aligned to the Greengenes database (http://greengenes.secondgenome.com/) using assign_taxonomy.py program (http://qiime.org/scripts/assign_taxonomy.html), with a minimum support threshold of 80%. Alpha diversity (number of OTUs, Shannon’s index, Chao1, Phylogenetic diversity_whole tree) and relative abundance of different taxa were calculated by alpha_diversity.py (http://qiime.org/scripts/alpha_diversity.html) included in the QIIME. Using the Vegan package (Version 2.4.0, Department of Statistics, Iowa State University, IA, USA), two different complementary analyses, ANOSIM and MRPP, were used to determine the significant differences in faecal microbiota in response to *M. oleifera* alternative diets.

### Sequencing data submission

All sequencing raw datasets have been deposited in the National Center for Biotechnology Information (NCBI) Sequence Read Archive (SRA) database (https://www.ncbi.nlm.nih.gov/sra/) with the BioProject accession number PRJNA351736. The sequencing files of 30 different faecal samples were numbered with accession numbers from SRR4639749 to SRR4639778.

### Statistical analysis

Data for nutrient digestibility, milk traits and faecal SCFAs of dairy cows were analysed using a one-way analysis of variance (ANOVA) and a protected Least Significant Difference Test (LSD) between groups by SPSS 19.0 (Chicago, IL, USA). The correlation analyses of genus taxa with the apparent digestibility, milk traits and faecal SCFAs were tested by the cor function (x, y, use = “p”) (http://127.0.0.1:11153/library/stats/html/cor.html), and illustrated with a function-labelled Heatmap (Matrix, xLabels, yLabels) in the R package WGCNA (http://127.0.0.1:11153/library/stats/html/cor.html). Metastat test^17^ was performed using the R software to determine the differences in the microbial composition (relative abundance) of the faecal microbiome.

## Additional Information

**How to cite this article:** Sun, J. *et al*. Characterization of faecal microbial communities of dairy cows fed diets containing ensiled *Moringa oleifera* fodder. *Sci. Rep.* **7**, 41403; doi: 10.1038/srep41403 (2017).

**Publisher's note:** Springer Nature remains neutral with regard to jurisdictional claims in published maps and institutional affiliations.

## Supplementary Material

Supplementary Information

Supplementary Table S3

Supplementary Table S4

Supplementary Table S6

## Figures and Tables

**Table 1:** Significant differences in community structure in the faecal microbiota of dairy cows among the three groups.

Group	Anosim analysis		MRPP analysis			
	R-value	P-value	A-value	Observed delta	Expected delta	P-value
L-H	0.048	0.167	0.006	0.321	0.323	0.207
C-H	0.224	0.024	0.036	0.325	0.337	0.018
C-L	0.138	0.069	0.022	0.326	0.334	0.030

Note: C, no *M. oleifera* diet; L, low *M. oleifera* diet; and H, high *M. oleifera* diet. Anosim, analysis of similarity; MRPP, multiresponse permutation procedure. The R-value ranged from −1 to 1, and an R-value > 0 that showed the significant differences in the between-group population compared with those in the within-group population, as well as A-value (analysed by MRPP, multiresponse permutation procedure). The *P*-values indicated the significant differences at the levels of *P* < 0.05 or *P* < 0.01.

**Table 2:** Nutrient apparent digestibility, milk yield, and composition in dairy cows fed *M. oleifera* silage.

Item	Group (mean values ± standard error)		
	C	L	H
Dry matter digestibility (%)	67.087 ± 0.4345^A^	63.898 ± 0.4431^B^	63.580 ± 0.3602^B^
Crude protein digestibility (%)	61.063 ± 0.6309	61.650 ± 0.6700	60.295 ± 0.6603
Milk yield (kg/d)	17.735 ± 0.5597	17.859 ± 0.5692	15.764 ± 0.2045
Milk fat (%)	4.824 ± 0.2575	4.706 ± 0.2832	4.947 ± 0.2014
Milk protein (%)	4.256 ± 0.2009	4.138 ± 0.1652	4.195 ± 0.1507
Lactose (%)	4.836 ± 0.0699	4.953 ± 0.0430	4.851 ± 0.0700
Somatic cell count (×10^3^/mL)	215.625 ± 55.01	214.583 ± 65.66	257.200 ± 69.82

Note: C, no *M. oleifera* diet; L, low *M. oleifera* diet; and H, high *M. oleifera* diet. Treatments with different capital letters were significantly different at *P* < 0.01.

**Table 3:** Quantitative analysis of SCFAs in the faeces of dairy cows among the three groups.

Item	Group (mean values ± standard error)		
	C	L	H
Acetic acid	5.8336 ± 0.1379^a^	5.3759 ± 0.2081^ab^	5.0819 ± 0.2489^b^
Propionic acid	3.6385 ± 0.2041^a^	3.1545 ± 0.1433^b^	3.0663 ± 0.0724^b^
Butyric acid	1.9914 ± 0.0544	1.8163 ± 0.1062	1.7105 ± 0.1236
Isovaleric acid	0.0802 ± 0.0018^A^	0.0654 ± 0.0307^B^	0.0539 ± 0.0233^C^
Valeric acid	0.2173 ± 0.0152	0.2152 ± 0.0141	0.1937 ± 0.0099

Note: C, no *M. oleifera* diet; L, low *M. oleifera* diet; and H, high *M. oleifera* diet. Treatments with different lowercase letters were significantly different at *P* < 0.05, and different capital letters identified a significant level at *P* < 0.01.
