# Supplementary material for: Characterization of faecal microbial communities of dairy cows fed diets containing ensiled Moringa oleifera fodder
Source: Sci Rep. 2017 Jan 30;7:41403. doi: 10.1038/srep41403 (PMC5278366; doi:10.1038/srep41403)
Supplement: Supplementary Information [file srep41403-s1.doc]

**Characterization of** **faecal** **microbial communities** **of dairy cows fed diets containing** **ensiled *Moringa oleifera*** **fodder**

Jiajie Sun ^1^, Bin Zeng ^1^, Zujing Chen ^2^, Shijuan Yan ^3^, Wenjie Huang ^3^, Baoli Sun ^1^, Qian He ^2^, Xiaoyang Chen ^2^, Ting Chen ^1^, Qingyan Jiang^1^, Qianyun Xi ^1,*^, Yongliang Zhang ^1,2 *^

1. College of Animal Science, Guangdong Provincial Key Laboratory of Agro-Animal Genomics and Molecular Breeding, National Engineering Research Center for Breeding Swine Industry, South China Agricultural University, Guangzhou, Guangdong 510642, China;
2. College of Forestry and Landscape Architecture, Guangdong Engineering ＆ Research Center for Woody Fodder Plants, South China Agricultural University, Guangzhou, Guangdong 510642, China;
3. Agro-biological Gene Research Center, Guangdong Academy of Agricultural Sciences, Guangzhou 510640, Guangdong Province, China.

Jiajie Sun and Bin Zeng contributed equally to this work.

*Correspondence and requests for materials should be addressed to Qianyun Xi or Yongliang Zhang.

Phone: +86-135 2780 3004

Fax: +86-020-8528 5931

E-mail: [zhangyl@scau.edu.cn](mailto:zhangyl@scau.edu.cn)

Table S1. Ingredients and nutrient contents of the experimental diets

| Item | C | L | H |
| --- | --- | --- | --- |
| Ingredients (% of Feed basis) |  |  |  |
| Concentrate mixes | 24.60 | 24.60 | 24.60 |
| Soybean hull | 11.18 | 11.18 | 11.18 |
| Beet | 5.59 | 5.59 | 5.59 |
| Whole cottonseed | 2.80 | 2.80 | 2.80 |
| Saccharose | 2.80 | 2.80 | 2.80 |
| Pineapple waste | 13.98 | 13.98 | 13.98 |
| Molasses | 1.68 | 1.68 | 1.68 |
| Oat hay | 4.19 | 4.19 | 4.19 |
| Chinese wildrye | 2.80 | 2.80 | 2.80 |
| Alfala hay | 6.99 | 5.24 | 3.49 |
| Maize straw silage | 23.41 | 11.71 | 0.00 |
| MO silage | 0.00 | 13.45 | 26.90 |
| Total | 100.00 | 100.00 | 100.00 |
| Nutrient contents(% of Dry matter) |  |  |  |
| NEL (MJ/Kg of Dry matter) | 6.25 | - | - |
| Dry matter (% of Wet) | 53.76 | 53.51 | 53.32 |
| Crude protein | 14.23 | 14.02 | 13.76 |
| Neutral detergent fiber | 45.32 | 45.18 | 45.05 |
| Acid detergent fiber | 27.76 | 28.09 | 28.26 |
| Ca | 0.85 | 0.85 | 0.84 |
| P | 0.48 | 0.48 | 0.49 |

Note: C, no *M. oleifera* diet; L, low *M. oleifera* diet; and H, high *M. oleifera* diet.

Table S2. Quality control and preprocessing of metagenomic datasets

| Sample Name | Raw PE | Effective Tags | Base (bp) | AvgLen (bp) | Q20 | Q30 | Effective (%) |
| --- | --- | --- | --- | --- | --- | --- | --- |
| C1 | 43093 | 38850 | 16046412 | 413.04 | 94.53% | 90.90% | 90.15 |
| C2 | 43737 | 38252 | 15881648 | 415.18 | 94.44% | 90.82% | 87.46 |
| C3 | 34706 | 32313 | 13287311 | 411.21 | 95.15% | 91.81% | 93.1 |
| C4 | 44977 | 41727 | 17175607 | 411.62 | 94.82% | 91.33% | 92.77 |
| C5 | 34912 | 30324 | 12543201 | 413.64 | 94.22% | 90.53% | 86.86 |
| C6 | 43723 | 37906 | 15737622 | 415.17 | 94.34% | 90.73% | 86.7 |
| C7 | 38798 | 33805 | 13977181 | 413.46 | 94.31% | 90.66% | 87.13 |
| C8 | 40774 | 35990 | 14917756 | 414.5 | 94.39% | 90.77% | 88.27 |
| L1 | 35198 | 30246 | 12500377 | 413.29 | 93.84% | 90.08% | 85.93 |
| L2 | 40845 | 36993 | 15431244 | 417.14 | 94.80% | 91.26% | 90.57 |
| L3 | 43786 | 36434 | 15074492 | 413.75 | 93.64% | 89.76% | 83.21 |
| L4 | 39165 | 34647 | 14335119 | 413.75 | 94.50% | 90.97% | 88.46 |
| L5 | 36304 | 30578 | 12657200 | 413.93 | 93.90% | 90.16% | 84.23 |
| L6 | 42093 | 37944 | 15718577 | 414.26 | 94.55% | 90.98% | 90.14 |
| L7 | 38945 | 34218 | 14181190 | 414.44 | 94.76% | 91.33% | 87.86 |
| L8 | 51142 | 38763 | 16068195 | 414.52 | 92.44% | 88.25% | 75.79 |
| L9 | 35095 | 31292 | 12992893 | 415.21 | 94.64% | 91.07% | 89.16 |
| L10 | 45854 | 36413 | 15013588 | 412.31 | 93.33% | 89.37% | 79.41 |
| L11 | 53251 | 40776 | 16937249 | 415.37 | 92.33% | 88.06% | 76.57 |
| L12 | 54137 | 41418 | 17225870 | 415.9 | 92.75% | 88.55% | 76.51 |
| H1 | 44249 | 36572 | 15080407 | 412.35 | 93.62% | 89.77% | 82.65 |
| H2 | 54732 | 43732 | 18093457 | 413.73 | 92.92% | 88.87% | 79.9 |
| H3 | 58766 | 42307 | 17580089 | 415.54 | 92.22% | 87.88% | 71.99 |
| H4 | 47834 | 42692 | 17648720 | 413.4 | 94.56% | 91.04% | 89.25 |
| H5 | 41705 | 36697 | 15162854 | 413.19 | 94.33% | 90.69% | 87.99 |
| H6 | 37520 | 32927 | 13617777 | 413.57 | 94.29% | 90.57% | 87.76 |
| H7 | 48977 | 39988 | 16668427 | 416.84 | 93.49% | 89.57% | 81.65 |
| H8 | 42568 | 38037 | 15728557 | 413.51 | 94.66% | 91.17% | 89.36 |
| H9 | 43435 | 36781 | 15221510 | 413.84 | 94.31% | 90.65% | 84.68 |
| H10 | 39235 | 35341 | 14624757 | 413.82 | 94.67% | 91.15% | 90.08 |

Note: Raw PE, original sequencing data; Effective Tags, final valid reads; Base, the total base number of effective tags; AvgLen, the average length of effective tags; Q20, the percentage of effective tags with the Q value of each base ≥ 20 (sequencing error rate ≤ 1%); Q30, the percentage of effective tags with the Q value of each base ≥ 30 (sequencing error rate ≤ 0.1%); and Effective, the percentage of effective tags in original sequencing reads.

**Table S3.** Sequence composition of each sample at each level in the Greengenes database

**Table S4.** Thirteen phyla-level and the top 35 genus-level taxa identified in the faeces of dairy cows

Table S5. Microbial diversity indices in the faecal microbiota of dairy cows among three groups

| Diversity Index | C | L | H | *P* vaule |
| --- | --- | --- | --- | --- |
| Shannon | 7.54 ± 0.07 ^a^ | 7.32 ± 0.10 | 7.21 ± 0.11 ^b^ | 0.098 |
| Chao1 | 760.84 ± 14.39 | 714.79 ± 27.41 | 703.30 ± 29.55 | 0.331 |
| Observed OUTs | 717.75 ± 14.44 | 665.92 ± 26.42 | 656.50 ± 28.24 | 0.251 |
| PD_whole_tree | 41.91 ± 0.45 | 39.32 ± 1.18 | 39.30 ± 1.20 | 0.212 |

Note: the data show the mean ± Standard Deviation, and treatments with different letters were significantly different at *P*<0.05. C represents the no *M. oleifera* group (N=8); L represents the low *M. oleifera* group (N=12); H represents the high *M. oleifera* group (N=10).

**Table S6.** Significant differences between the C and H groups identified at phylum-taxa level and OTU-taxa level

Table S7. Pearson correlation analysis of Phyla taxa with the apparent digestibility and milk traits, R value (P-value)

| Taxonomy | DM | CP | My | Mf | Mp | Ml | Sc |
| --- | --- | --- | --- | --- | --- | --- | --- |
| *Firmicutes* | 0.259 (0.167 ) | -.0380 (0.858) | 0.083 (0.695) | -0.079 (0.708) | -0.107 (0.612) | 0.362 (0.075) | 0.547 (0.005)^**^ |
| *Bacteroidetes* | -0.233 (0.215) | -0.291 (0.158) | -0.275 (0.183) | 0.169 (0.419) | 0.087 (0.679) | -0.251 (0.226) | -0.497 (0.011)^*^ |
| *Spirochaetes* | -0.191 (0.312) | 0.320 (0.118) | -0.084 (0.689) | -0.026 (0.900) | 0.178 (0.396) | -0.503 (0.010)^*^ | -0.338 (0.098) |
| *Proteobacteria* | -0.031 (0.871) | 0.14 (0.504) | 0.345 (0.091) | -0.081 (0.701) | -0.143 (0.495) | 0.235 (0.258) | 0.109 (0.604) |
| *Verrucomicrobia* | 0.277 (0.138) | 0.234 (0.261) | 0.040 (0.850) | -0.066 (0.754) | 0.156 (0.457) | -0.033 (0.875) | -0.302 (0.142) |
| *Tenericutes* | -0.142 (0.454) | -0.398 (0.049)^*^ | 0.189 (0.365) | -0.148 (0.480) | 0.058 (0.783) | 0.160 (0.445) | -0.164 (0.434) |
| *Actinobacteria* | 0.146 (0.440) | -0.311 (0.130) | -0.340 (0.096) | 0.392 (0.053) | -0.072 (0.733) | 0.077 (0.715) | 0.281 (0.174) |
| *Euryarchaeota* | 0.187 (0.321) | 0.009 (0.965) | 0.310 (0.132 ) | -0.188 (0.368) | -0.218 (0.296) | 0.211 (0.312) | -0.071 (0.737) |
| *Cyanobacteria* | 0.233 (0.216) | 0.291 (0.159) | 0.050 (0.812) | -0.014 (0.948) | 0.098 (0.640) | 0.016 (0.939) | -0.196 (0.348) |
| *Fibrobacteres* | 0.470 (0.009)^**^ | 0.349 (0.088) | -0.156 (0.457) | -0.097 (0.643) | 0.061 (0.773) | 0.035 (0.867) | -0.295 (0.152) |
| *TM7* | -0.050 (0.791) | 0.025 (0.906) | -0.034 (0.871 ) | 0.163 (0.435) | -0.061 (0.773) | -0.023 (0.913) | 0.163 (0.435) |
| *Elusimicrobia* | 0.224 (0.233) | -0.118 (0.575) | -0.259 (0.211) | 0.112 (0.594) | 0.064 (0.760) | -0.152 (0.469) | -0.080 (0.703) |
| *Lentisphaerae* | 0.172 (0.362 ) | -0.091 (0.664) | 0.140 (0.504) | 0.216 (0.301) | 0.096 (0.649) | 0.039 (0.853) | 0.133 (0.528) |

Note: * The correlation is significant at a level of 0.05; ** the correlation is significant at a level of 0.01. DM: dry matter digestibility; CP: crude protein digestibility; My: milk yield; Mf: milk fat; Mp: milk protein; Mi: lactose, Sc: somatic cell count.


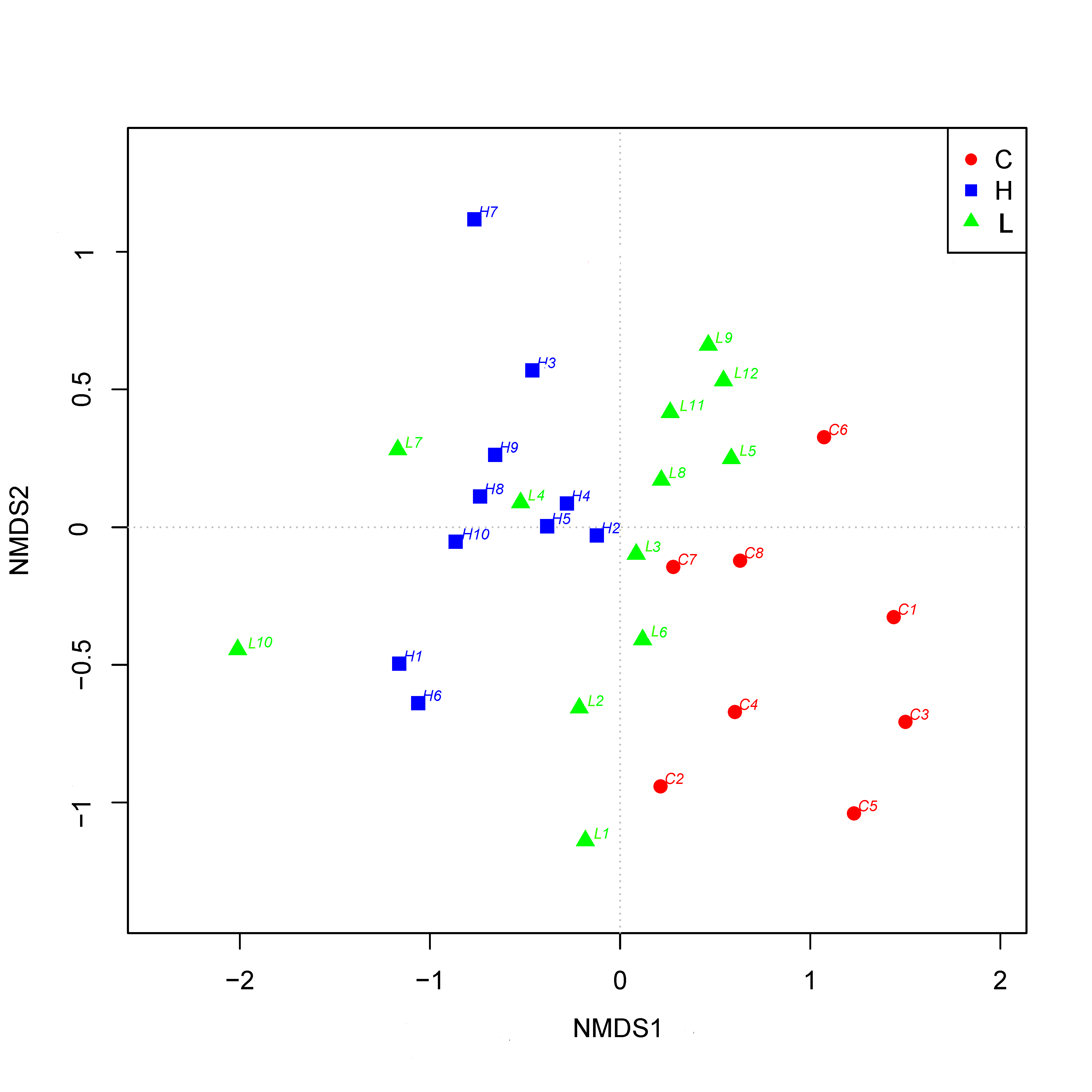


Figure S1. Distinct differences in the community structure of the faecal microbiota of dairy cows among three groups

Note: Non-Metric Multi-Dimensional Scaling (NMDS) analysis was performed by metaMDS function in the R package Vegan v2.4-1. NMDS = 0.1784, which is t less than 0.2, implying that the result was reliable. C represents the no *M. oleifera* group (N=8); L represents the low *M. oleifera* group (N=12); H represents the high *M. oleifera* group (N=10).
